# Investigating the Anti-inflammatory Effect of Allopurinol on the Prevention of Prostate Cancer

**DOI:** 10.7759/cureus.40058

**Published:** 2023-06-06

**Authors:** Marc Ganz, Christopher Alessandro, Menachem Jacobs, Daniel Miller, Yehuda Gejerman, Frederick Okoye, Scott Jamieson, Andrew Winer

**Affiliations:** 1 Public Health Sciences, State University of New York Downstate Health Sciences University, Brooklyn, USA; 2 Internal Medicine, Icahn School of Medicine at Mount Sinai, Queens Hospital Center, Queens, USA; 3 Internal Medicine, New York Medical College, Valhalla, USA; 4 Urology, State University of New York Downstate Health Sciences University, Brooklyn, USA

**Keywords:** logistic regression analysis, prostate cancer, anti-inflammatory drugs, allopurinol, gout

## Abstract

Introduction

Gout is a form of arthritis that arises from the accumulation of uric acid in the bloodstream. Allopurinol, a medication that reduces uric acid levels, has also been shown to have anti-inflammatory effects. Research in this area seems to have mixed results. Furthermore, limited research has examined the relationship between gout treated with Allopurinol and its possible protective factors against prostate cancer. The purpose of this study was to examine the relationship between Allopurinol use and prostate cancer, controlling for demographic and metabolic factors.

Methods

Information was collected from the National Health and Nutrition Examination Survey (NHANES) dataset of the Centers for Disease Control and Prevention (CDC). Logistic regression analysis was employed to establish the correlation between the usage of Allopurinol and the occurrence of prostate cancer while considering variables such as weight, hypertension, hyperlipidemia, race, educational level, and marital status. The research received approval from the review board of the Physician's Journal of Medicine.

Results

We found no significant association between Allopurinol use and prostate cancer, controlling for covariates. Age was found to have a positive association with prostate cancer. Marriage was found to have a negative association with prostate cancer.

Conclusion

The results of this study did not find a significant association between Allopurinol use and the risk of prostate cancer. However, this study adds to the limited body of research examining the relationship between gout, Allopurinol, and prostate cancer and suggests that further research is needed in this area. Overall, while Allopurinol has been shown to have anti-inflammatory effects and is used to treat gout, its use does not appear to have a significant impact on the risk of developing prostate cancer.

## Introduction

Gout is a form of arthritis that arises from the accumulation of uric acid in the bloodstream. This buildup leads to the formation of small crystals that deposit in the joints, causing pain, swelling, redness, and warmth. Over time, gout can cause joint damage, erosions, and tophi to form. Gout is often treated with medication that reduces uric acid production and removes it from the body, along with pain relief and anti-inflammatory drugs. Allopurinol is a medication that is used to reduce the levels of uric acid in the body. In addition to its role in reducing uric acid levels, Allopurinol has been shown to have anti-inflammatory effects [1,2].

Some studies suggest that the use of anti-inflammatory medications may be associated with a lower risk of prostate cancer or with improved outcomes for men with prostate cancer [3-6]. However, other studies have found no significant relationship between anti-inflammatory medication use and prostate cancer occurrence and mortality [7,8]. This raises the question as to whether Allopurinol may decrease the risk of prostate cancer either in its uric acid-lowering effects or its anti-inflammatory effects.

There is limited research examining the relationship between gout treated with Allopurinol and its possible protective factors against prostate cancer. Some studies have shown that gout may be associated with prostate cancer [9-11]. One study even identified a potential mechanism of the relationship between gout and prostate cancer demonstrating that a change in plasma or intracellular urate levels has an impact on prostate cancer cell growth [12]. This indicated that the use of uric acid-lowering agents, such as Allopurinol, would potentially be therapeutic in the prevention of prostate cancer.

However, though gout was shown to be correlated to prostate cancer [11], the definition of gout was those treated with Allopurinol. This indicates that while gout may be a risk factor for prostate cancer, treatment with Allopurinol did not mitigate this risk. Additionally, previous studies have found no clear association between Allopurinol use and prostate cancer risk [13,14].

In light of the above, there is much need to investigate the relationship between Allopurinol and prostate cancer. The objective of this study was to investigate the correlation between the utilization of Allopurinol and prostate cancer, taking into account demographic variables, such as age, race, and BMI, as well as factors related to gout and metabolic syndrome, including heart disease, hypertension, and diabetes.

## Materials and methods

Data were collected from the National Health and Nutrition Examination Survey (NHANES) dataset of the Centers for Disease Control and Prevention (CDC). This survey contained 3,030 participants. Questionnaire data from March 2017 to March 2020, specifically categorized as "Medical Conditions" and "Prescription Medications," were gathered. The study underwent submission and approval by the Institutional Review Board of the Physician's Journal of Medicine in Queens, New York, United States, on March 15, 2023 (Approval number: 2303F15). Logistic regression analysis was conducted to investigate the association between the usage of Allopurinol and the history of prostate cancer while controlling for variables such as weight, hypertension, hyperlipidemia, race, educational level, and marital status. The primary outcome variable in this study was the presence of a prostate cancer history, determined by a positive response to the question "Ever told you had cancer or malignancy," with "prostate" as the specific response to the subsequent question "What kind was it?" This was coded as True for "Yes" and False for "No." The predictor variable utilized was Allopurinol usage, assessed through the response to the question "In the past 30 days, have you used or taken medication for which a prescription is needed?" A response of "Allopurinol" was coded as True. Covariates, including BMI, age, race, educational level, and marital status, were also examined. Furthermore, other factors associated with both prostate cancer and inflammation, such as blood pressure and low-density lipoprotein, were controlled for in the analysis.

Statistical analysis

To assess the correlation between the predictor and outcome variables mentioned earlier, a multivariate logistic regression analysis was conducted to examine the association between the utilization of Allopurinol and the presence of a prior prostate cancer diagnosis. The variables taken into account in the analysis encompassed but were not limited to weight, age, race, educational level, and marital status. Calculations were performed to determine the adjusted odds ratios (OR), coefficients of association (B), and confidence intervals (CI). All analyses were performed using IBM SPSS Statistics for Windows, Version 28.0 (Released 2021; IBM Corp., Armonk, New York, United States).

## Results

Our study found that there was no significant association between Allopurinol use and prostate cancer, OR (odds ratio) = 1.764, CI (confidence interval) = -0.938 - 2.075. Other variables were found to be significant. Age was found to have a positive association with prostate cancer, meaning higher age was associated with a higher rate of prostate cancer, as previously well-established. Being married was found to have a negative association with prostate cancer, meaning being married was associated with a lower rate of prostate cancer (Tables 1, 2). This too is consistent with prior literature [15,16].

**Table 1:** Demographics of patients with prostate cancer

Variable	History of Prostate Cancer N (%)	No History of Prostate Cancer N (%)
Race/Hispanic Origin		
Mexican American	3 (5.00%)	57 (95.00%)
Other Hispanic	9 (14.06%)	55 (85.94%)
White	84 (14.05%)	514 (85.95%)
Black	45 (24.19%)	141 (75.81%)
Asian	4 (8.16%)	45 (91.84%)
Education Level		
Less than 9th grade	6 (10.71%)	50 (89.29%)
9-11th grade	14 (13.59%)	89 (86.41%)
High school graduate/GED or equivalent	42 (18.67%)	183 (81.33%)
Some college/AA degree	47 (13.66%)	297 (86.34%)
College graduate or above	40 (14.60%)	234 (85.40%)
Marital Status		
Married	93 (16.85%)	459 (83.15%)
Widowed/Divorced/Separated	49 (13.61%)	311 (86.39%)
Never married	7 (7.95%)	81 (92.05%)

**Table 2:** Correlation coefficients of variables and prostate cancer BMI: body mass index; SBP: systolic blood pressure; DBP: diastolic blood pressure; LDL: low-density lipoprotein

Variable	OR	B	Lower 95% CI	Upper 95% CI	p-value
Allopurinol	1.764	0.568	-0.938	2.075	0.459
Age	1.076	0.074	0.025	0.123	0.003
BMI	0.961	-0.04	-0.099	0.02	0.188
SBP	1	0	0	0	0.797
DBP	1	0	0	0	0.68
LDL	1	0	-0.000053	0	0.394
Race	1.713	0.538	-0.275	1.351	0.194
Education Level	0.849	-0.164	-0.475	0.147	0.302
Marriage	2.549	0.936	0.107	1.764	0.027

In summary, the study did not find a significant association between Allopurinol use and prostate cancer. However, we found that higher age was associated with a higher rate of prostate cancer and being married was associated with a lower rate of prostate cancer.

## Discussion

The data regarding Allopurinol use and its potential effects on prostate cancer incidence are conflicting. The reason for this is that it is known that Allopurinol has anti-inflammatory effects [1,2]. Some studies suggest that anti-inflammatory medications may be associated with a lower risk of prostate cancer [3,4].

However, other studies have found no significant relationship between anti-inflammatory medication use and prostate cancer occurrence and mortality [7,8]. For this reason, our study aimed to investigate the relationship between Allopurinol use and prostate cancer. We controlled for demographic factors such as age, race, marital status, and BMI. We also controlled for factors well-established to be associated with gout and metabolic syndrome since these factors would likely be an underlying cause of prostate cancer and mask the relationship between Allopurinol and prostate cancer alone [17].

Our findings, which showed no significant association between Allopurinol use and prostate cancer occurrence after adjusting for covariates, are consistent with previous research on this topic [18]. However, some previous studies have suggested a potential link between Allopurinol use and a reduced risk of prostate cancer [19,20]. It is possible that the anti-inflammatory effects of Allopurinol are not sufficient to impact prostate cancer risk or that other factors may be involved in the relationship between gout and prostate cancer.

It is important to note that our study has some limitations. The data used in our analysis is based on self-reported information, which may not always be accurate. In addition, the sample size of our study is relatively small, which may limit the generalizability of our findings. In addition, although the study controlled for several confounding variables, there may be other unmeasured factors that could affect the relationship between Allopurinol use and prostate cancer occurrence. Lastly, the study was observational and therefore cannot establish causality. Despite these limitations, this investigation contributes to the existing literature on the relationship between Allopurinol use and prostate cancer by providing further evidence on this topic.

## Conclusions

In conclusion, our study did not find a significant relationship between Allopurinol use and prostate cancer risk. While previous research has suggested that gout may be associated with prostate cancer, our results indicate that treatment with Allopurinol may not be sufficient to mitigate this risk.

Further research is needed to investigate the potential link between gout and prostate cancer, as well as the potential benefits of other medications used to treat gout in relation to prostate cancer risk. Our study highlights the importance of considering multiple factors when examining the relationship between medications and cancer risk, including demographic and health-related factors that may impact this relationship.
